# Supplementary material for: The Anthocyanin Accumulation Related ZmBZ1, Facilitates Seedling Salinity Stress Tolerance via ROS Scavenging
Source: Int J Mol Sci. 2022 Dec 17;23(24):16123. doi: 10.3390/ijms232416123 (PMC9783181; doi:10.3390/ijms232416123)
Supplement: Supplementary file 1 [file ijms-23-16123-s001.zip › ijms-2076309-supplementary.pdf]

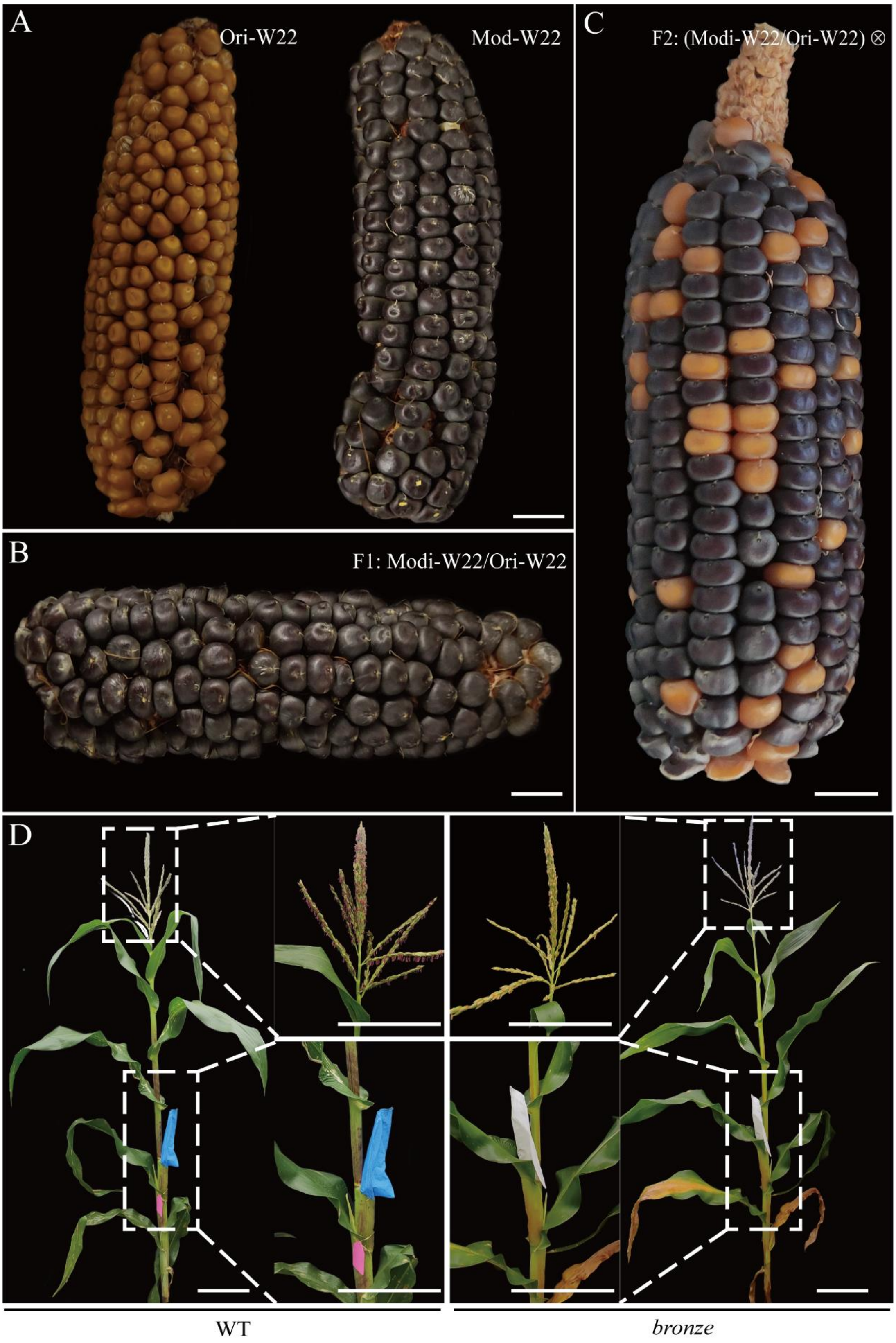

Figure S1. Phenotypic analysis of plants and ears from WT and *bronze* mutant. (**A**) An original W22 ear (left) and a modified W22 ear (right). Bar = 1 cm; (**B**) An F_1 ear from a Modi-W22/Ori-W22 plant. Bar = 1 cm; (**C**) An F_2 ear from a Modi-W22/Ori-W22 F1 plant. Bar = 1 cm; (**D**) Phenotypic observation of mature plants from WT and *bronze* mutant. Bar = 10 cm.

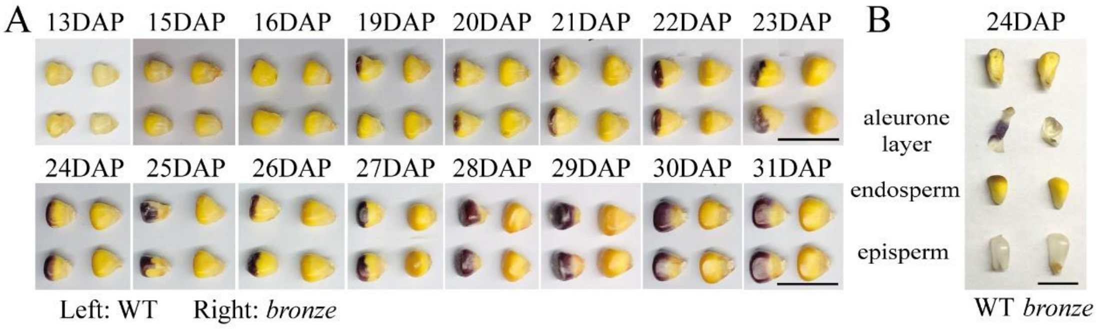

Figure S2. Pigments movement of developing seeds from WT and *bronze* at stages of 13 to 31 DAP. (**A**) Developing seeds of WT and *bronze* mutant from 13 to 31 DAP. Bar = 1 cm; (**B**) Separated parts of developing seeds (24 DAP) by longitudinal section, including WT and *bronze* whole seeds, the separated aleurone layer, endosperm and the seed coat (pericarp). Bar = 1cm.

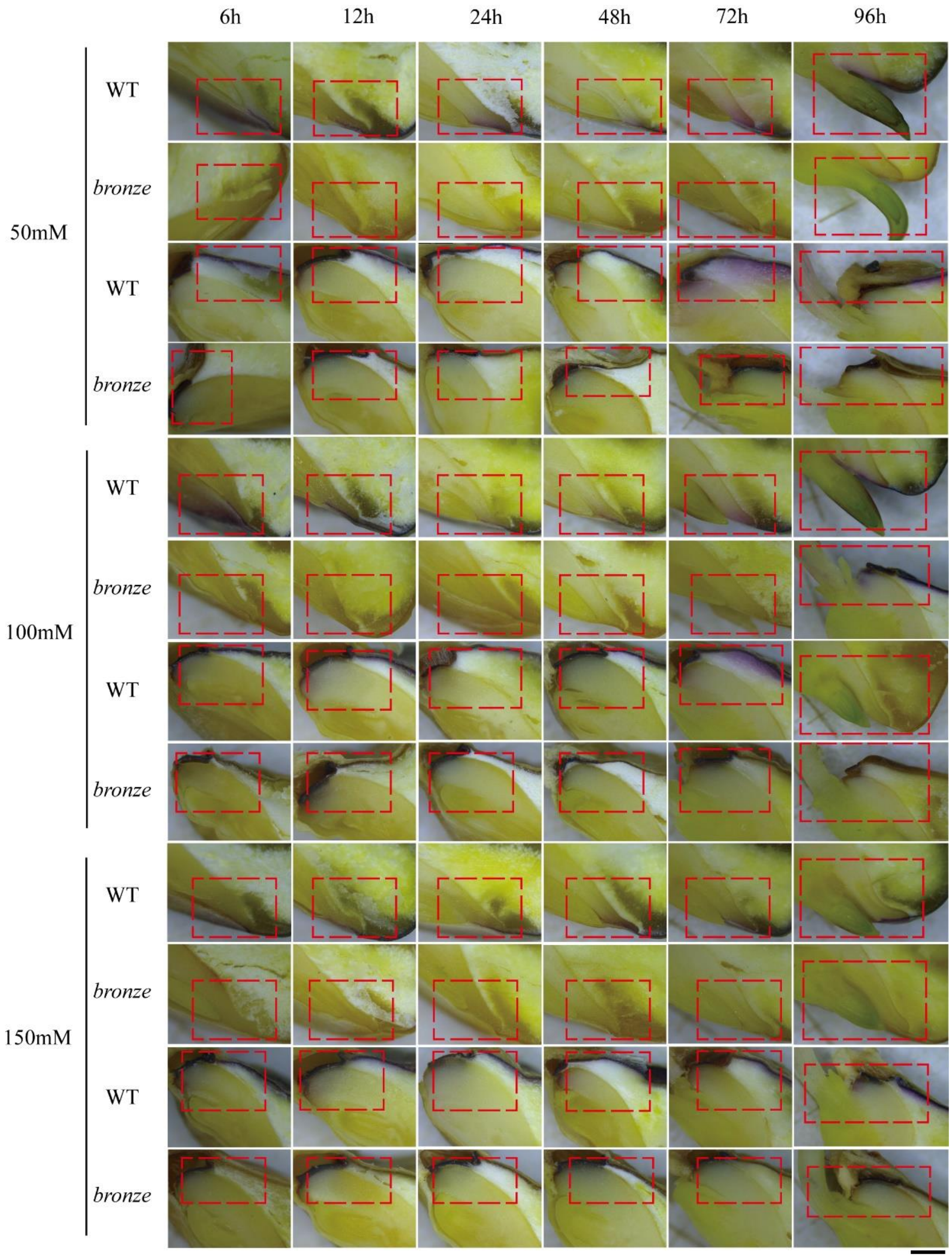

Figure S3. The correlation of anthocyanin movement and embryogenic cell division during seed germination under salinity stress. The pigments movement was observed from longitudinal sections of WT and *bronze* seeds at 6, 12, 24, 48, 72 and 96 hrs germination under 50 mM, 100 mM and 150 mM NaCl treatments. Red dashed rectangles highlight the location of anthocyanin accumulation. Bar=1 mm.

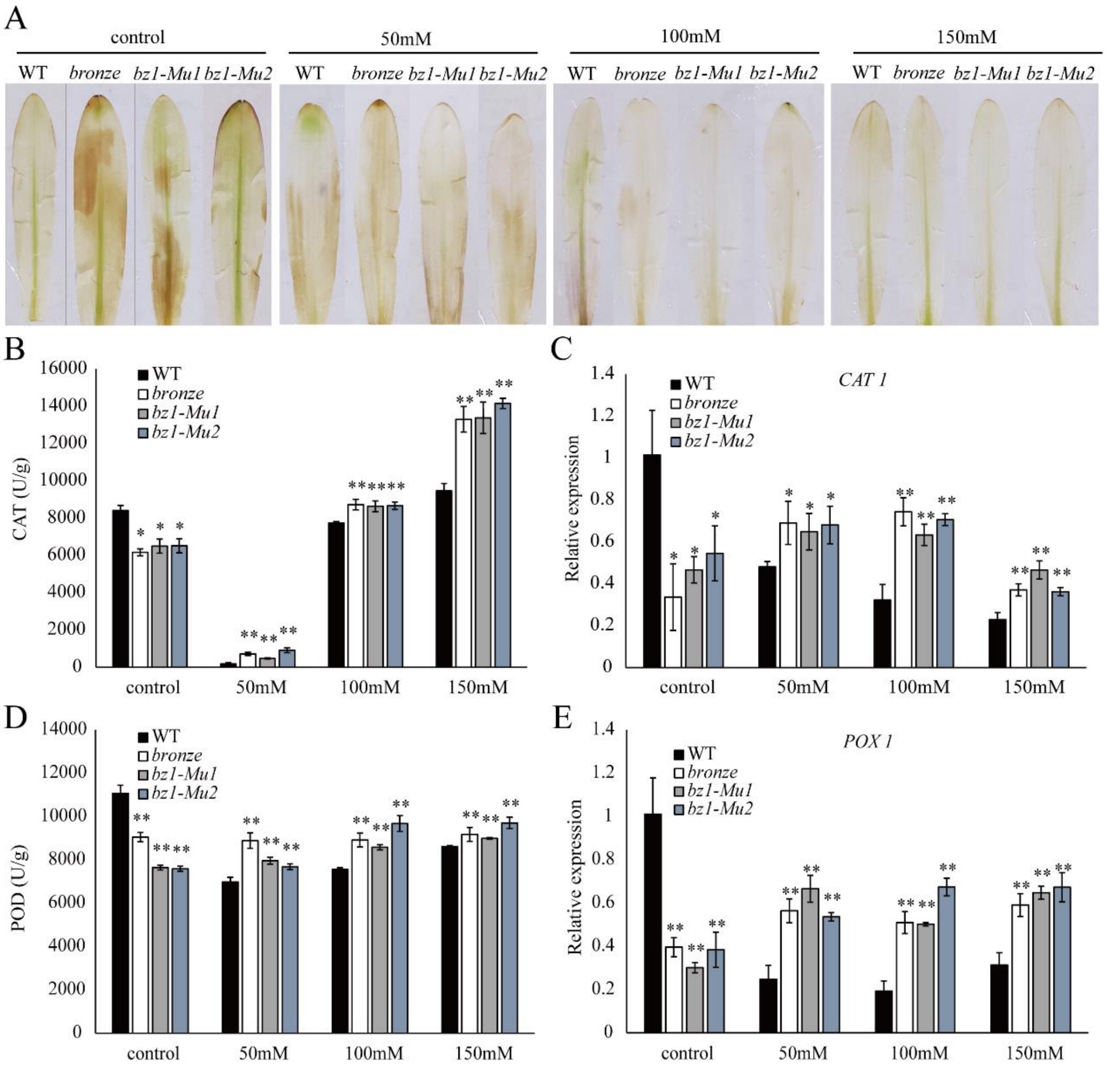

Figure S4. Antioxidant activity conferred by ZmBZ1 was independent of hydrogen peroxide. DAB staining (**A**), CAT activity (**B**), POD activity (**D**), *CAT1* and *POD1* expression (**C, E**) of three bronze alleles and WT seedlings at 14 DAS before and after NaCl treatment. *GAPDH* was used as an internal control. Values are shown as means ± SE, n = 3. Asterisks indicate significant differences relative to the WT (Student's t-test: $^*P < 0.05$; $^{**}P < 0.01$).

Table S1 The segregation ratio of the F_2 ears and allelic test via Chi-square test.

Material	Ear	Number of WT kernels	Number of mutant kernels	Chi-square value	$\chi 2$ (0.05)
Modi-W22/ Ori-W22 F_2	4191-3	57	15	0.46	3.84
	4191-11	75	23	0.05	
	4191-13	82	32	0.42	
bz1/+ F_2	3442-9	127	52	1.36	
	3443-6	280	80	1.34	
	3443-14	185	74	1.58	
bz1-Mu1/+ F_2	3447-13	233	62	2.29	
	3447-17	201	73	0.31	
	3448-6	238	75	0.13	
bz1-Mu2/+ F_2	3446-14	167	44	1.72	
	3446-15	210	67	0.06	
	3446-22	143	54	0.49	

Table S2 Primer sequences used in gene mapping, expression and plasmids constructing.

Primer Name	Sequence (5' to 3')	Use for
MF	CCACGCTCTCGTTCCTCTC	Identification of *Mu* insertion site and genotype of *ZmBZ1* in WT, *bronze*, *bz1-Mu1* and *bz1-Mu2*
MR	GCAGTTGGGCAGGATCTC	
MF1	GTGGCACTCAACACGTTCC	
MF2	TCACGCGCGACAGACTATCT	
MuTIR	AGAGAAGCCAACGCCAWCGCCTCYATTTCGTC	
GAPDH-qF	CCATCACTGCCACACAGAAAAC	Internal control for all qRT-PCR gene expression
GAPDH-qR	AGGAACACGGAAGGACATACCAG	
ZmBz1-qF	CGCACGTGTGGGGGTTC	qRT-PCR analysis of *ZmBZ1* gene expression
ZmBZ1-qR	ATCTCGACGAACCTGTCGAA	
DFR-qF	CACCTTCAGGTACAAGACGC	qRT-PCR analysis of *DFR* gene expression
DFR-qR	GCGCCAATCGTCGCCT	
ANS-qF	CTGGGTCGTCTTCTGCGA	qRT-PCR analysis of *ANS* gene expression
ANS-qR	TGCTTTTGCTTTGTGCTGCTG	
F3H-qF	CTGAGCAACGGCAGGTTCAA	qRT-PCR analysis of *F3H* gene expression
F3H-qR	GTACATCTCGGCGAAGGTGA	
CAT1-qF	GGCTGTCGTGAGAAGTGCAT	qRT-PCR analysis of *CAT1* gene expression
CAT1-qR	ACTGGGACCAGTAGGAGATCCA	
POX1-qF	GCTCTTCTTCTCCGACAACCA	qRT-PCR analysis of *POX1* gene expression
POX1-qR	GTACGAACTCGGGTTGACGAC	
SOD3-qF	ACAAGGGTCTGGATGGGTGTG	qRT-PCR analysis of *SOD3* gene expression
SOD3-qR	AGGTAGTATGCATGTTCCCAGACAT	

MYB109-qF	AGAGCAACGTCACAGACACC	qRT-PCR analysis of *MYB109* gene expression
MYB109-qR	CCTGCTCATGTACTGCTGGC	
MYBR51-qF	ACAAGCTCGAAGCAACTCCA	qRT-PCR analysis of *MYBR51* gene expression
MYBR51-qR	AGAGAGACGAACGGACGGA	
pGWC-BZ1:YFP-F	AGCAGGCTTTGACTTTATGGCGCCCGCCGACGGCGA	Construct subcellular localization vector
pGWC-BZ1:YFP-R	TGGGTCTAGAGACTTTTCCGCGCGACAGACTATCT	
BZ1-16318hGFP-F	TATCTCTAGAGGATCCATGGCGCCCGCCGACGGCGA	
BZ1-16318hGFP-R	TGCTCACCATGGATCCCGCGCGACAGACTATCT	
pAbAi-BZ1-F	CTTGAATTCGAGCTCGTCTCTTATTAAAGCACGCC	Y1H and Y2H analysis
pAbAi-BZ1-R	AGCACATGCCTCGAGGCCATCTTCGCGCGCTCAGG	
pGADT7-MYB109-F	GGAGGCCAGTGAATTCATGAGGAAACCGGAGTGCCC	
pGADT7-MYB109-R	CGAGCTCGATGGATCCTCATTCAACTTGGAAATCAA	
pGADT7-MYBR51-F	GGAGGCCAGTGAATTCATGGGTATTACATGCAGCCA	
pGADT7-MYBR51-R	CGAGCTCGATGGATCCTTACAACAAAGAGAGACGAA	
pGBKT7-MYB109-F	CATGGAGGCCGAATTCATGAGGAAACCGGAGTGCCC	
pGBKT7-MYB109-R	GCAGGTCGACGGATCCTCATTCAACTTGGAAATCAA	
pGWC-MYB109:YFP-F	AGCAGGCTTTGACTTTATGAGGAAACCGGAGTGCCC	BiFC analysis
pGWC-MYB109:YFP-R	TGGGTCTAGAGACTTTTCTTCAACTTGGAAATCAA	
pGWC-MYBR51:YFP-F	AGCAGGCTTTGACTTTATGGGTATTACATGCAGCCA	
pGWC-MYBR51:YFP-R	TGGGTCTAGAGACTTTTTCAACAAAGAGAGACGAA	
